# Genetic Code Expansion: A Brief History and Perspective

**DOI:** 10.1021/acs.biochem.1c00286

**Published:** 2021-07-01

**Authors:** Mia A. Shandell, Zhongping Tan, Virginia W. Cornish

**Affiliations:** † York Structural Biology Laboratory, University of York, Heslington, York YO10 5DD, U.K.; ‡ State Key Laboratory of Bioactive Substance and Function of Natural Medicines, Institute of Materia Medica, Chinese Academy of Medical Sciences and Peking Union Medical College, Beijing 100050, China; § Department of Chemistry, Columbia University, New York, New York 10027, United States; ∥ Department of Systems Biology, Columbia University, New York, New York 10027, United States

## Abstract

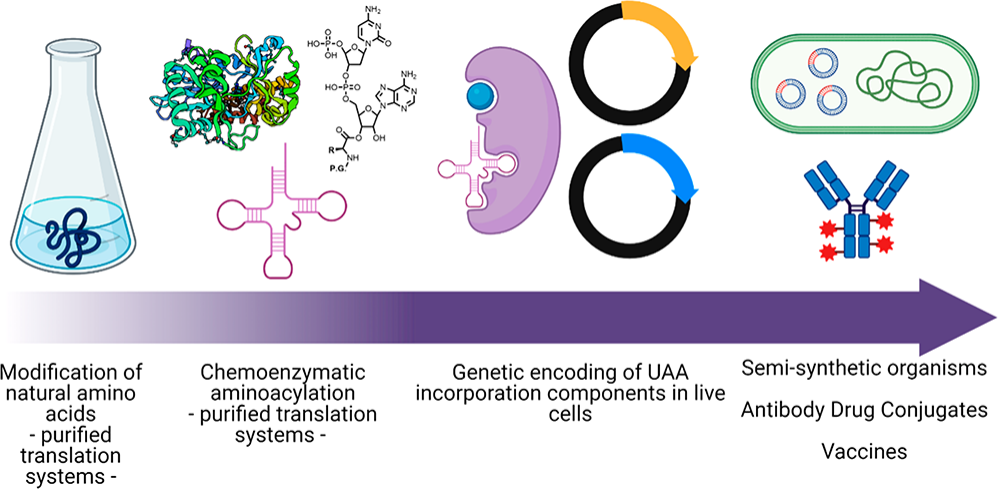

Since the establishment of site-specific mutagenesis of single amino acids to interrogate protein function in the 1970s, biochemists have sought to tailor protein structure in the native cell environment. Fine-tuning the chemical properties of proteins is an indispensable way to address fundamental mechanistic questions. Unnatural amino acids (UAAs) offer the possibility to expand beyond the 20 naturally occurring amino acids in most species and install new and useful chemical functions. Here, we review the literature about advances in UAA incorporation technology from chemoenzymatic aminoacylation of modified tRNAs to *in vitro* translation systems to genetic encoding of UAAs in the native cell environment and whole organisms. We discuss innovative applications of the UAA technology to challenges in bioengineering and medicine.

A grand challenge for the field of biochemistry is a general method for tailoring protein structure to address biological mechanism in the native cellular environment and to endow cells with new functions for future engineering applications. Advances in molecular biology in the 1970s made site-specific mutagenesis of single amino acids to probe protein function an everyday reality for researchers. Expanding the mutagenesis repertoire beyond the 20 naturally occurring amino acids in most species, unnatural amino acids (UAAs) enable site-specific installation of new and useful chemical functions, fluorescence, ligand binding, cross-linking, or photocaging, for example. There are still challenges to UAA mutagenesis being an everyday technique for biochemists. In this Perspective, we highlight the 2013 work of Chatterjee and Schultz, key historical papers in the field, and key perspective papers that illustrate future directions being charted by researchers in the field. This Perspective is not meant to stand in for comprehensive reviews published by researchers in the field.^1−4^ Furthermore, we acknowledge that there are several exciting technologies that have been developed over the past several decades for chemical modification of proteins; we speak to only the UAA technology here. Chatterjee et al. (Schultz)^5^ is a landmark study demonstrating a streamlined plasmid-based system for efficient multisite UAA incorporation in one target protein in live bacterial cells.

Chatterjee et al. (Schultz)^5^ integrated conceptual advances in orthogonal aminoacyl-tRNA synthetase (aaRS)/tRNA generation, multisite incorporation, and flexibility of codon usage in a minimalist, optimized system for incorporation of UAAs in living cells. UAAs are incorporated into proteins in live cells by bio-orthogonal aaRS enzymes evolved to bind the UAA and its unnatural tRNA but not interact with the naturally occurring amino acids or tRNAs (Figure 1). The unnatural tRNA recognizes a stop, quadruplet base pair, or frameshift codon such that this combination manipulates the cell’s endogenous translational machinery to incorporate the UAA into the target protein at the specific site of interest.

**Figure 1:**
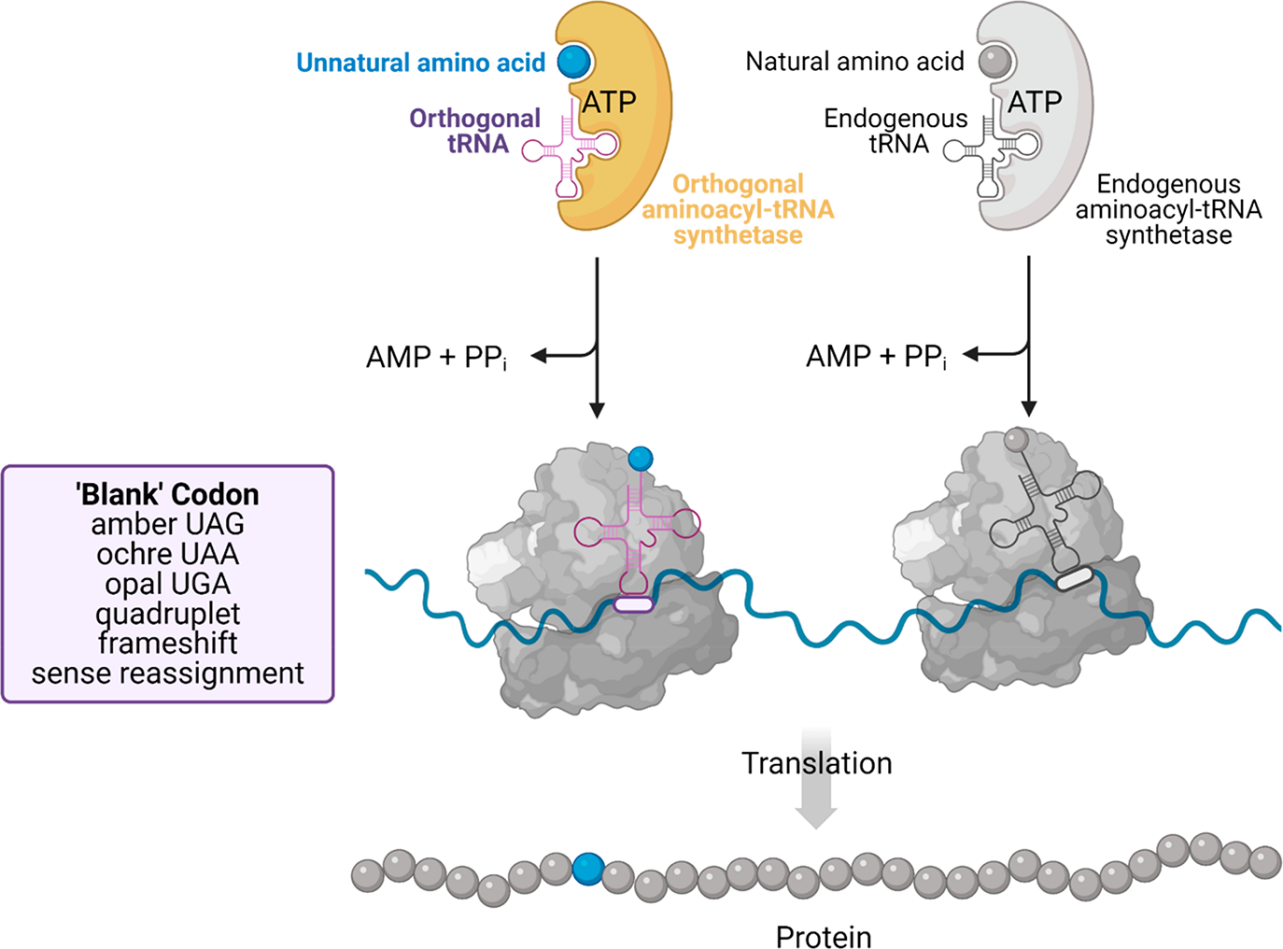
Unnatural amino acid (UAA) incorporation.

Chatterjee et al. (Schultz)^5^ advanced the field by streamlining multisite UAA incorporation into a simple *Escherichia coli* transformation with two plasmids. One plasmid pUltraII encoded one copy each of two orthogonal aaRS/suppressor tRNA pairs: amber (UAG) suppressing *Methanococcus jannaschii* tyrosyl (*Mj*Tyr)-derived aaRS/tRNA_CUA_ and optimized ochre (UAA) suppressing *Methanosarcina barkeri* pyrrolysyl (*Mb*Pyl)RS/*Methanosarcina mazei* pyrrolysyl (*Mm*Pyl)-tRNA_UUA_. The second plasmid encoded target protein green fluorescent protein (GFP) containing amber and ochre nonsense codons (GFP-3TAG-151TAA) to direct the incorporation of two unique UAAs into a single protein. This was previously intractable due to the requirement for multiple copies of aaRS or tRNA expression cassettes to incorporate a single UAA. The predecessor of pUltraII, pEVOL, encoded one copy of a *Mj*Tyr-derived optimized amber suppressor tRNA and two copies of *Mj*TyrRS to incorporate UAAs into GFP151TAG.^6,7^ With this construct in the presence of UAA *p*-azido-l-phenylalanine (pAzF), the suppression efficiency for a single amber codon reached approximately 80% of that of wild-type GFP.^7^ Incorporation of two UAAs, *p*-acetyl-l-phenylalanine (pAcF) and *N*_ε_-Boc-l-lysine (eBK), into GFP-3TAG-151TAA using pUltraII achieved 20–25% of wild-type GFP expression.^5^

Generality was shown by optimizing incorporation of these UAA pairs simultaneously into GFP using the amber and ochre suppressor aaRS/tRNAs: pAcF and azido-l-lysine (AzK), pAcF and eBK, pAzF, and eBK, and *O*-methyl-l-tyrosine (OMeY) and eBK (Figure 2). UAAs with click handles, for example, pAcF and AzK, were incorporated for dual labeling with dyes suitable for in-gel Förster resonance energy transfer (FRET). They applied the dual suppression system to label a nonfluorescent target, ketosteroid isomerase, with a FRET pair by incorporating acetyl and azido click handles and labeling with Alexa Fluor 488-hydroxylamine to label the ketone and Alexa Fluor 594 dibenzocyclooctynol to label the azide, postpurification.^5^

**Figure 2:**
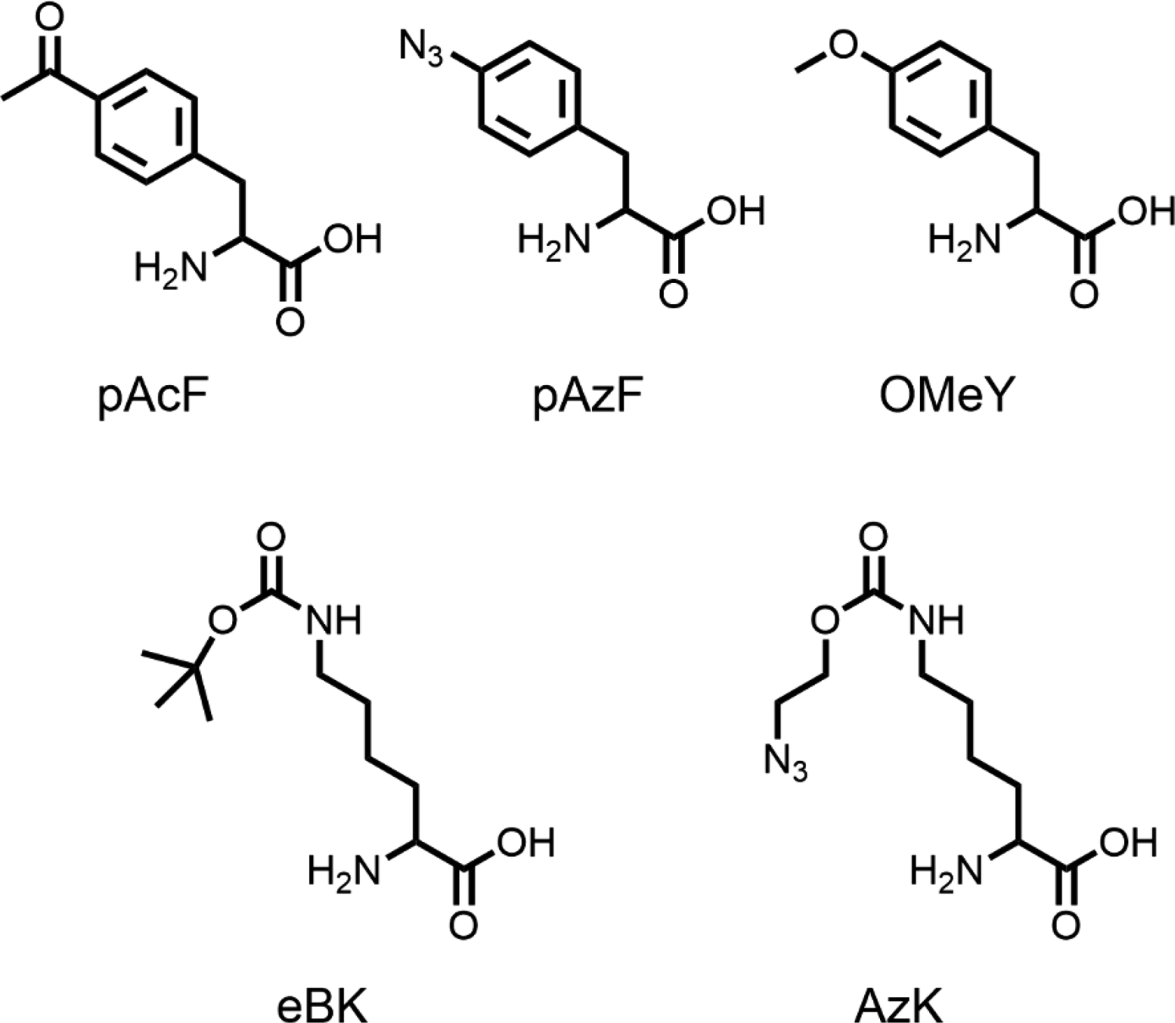
Chemical structures of UAAs incorporated into GFP-3TAG-151TAA in Chatterjee et al. (Schultz).^5^

## Early Research with UAAs

Researchers began exploring the possibility of using modified tRNAs to incorporate UAAs soon after the discovery of the tRNA adaptor.^8,9^ In fact, the tRNA adaptor hypothesis was proven by chemically reducing Cys-tRNA^Cys^ to Ala-tRNA^Cys^ and showing that Ala would then be incorporated in response to a poly-Cys template.^10^ In 1967, it was shown that the translational machinery could utilize d-Tyr-tRNA^Tyr^, prepared enzymatically by tyrosyl-tRNA synthetase (TyrRS), as a substrate.^11^ The flexibility of the ribosomal peptidyl transferase center (PTC) to unusual chemistry was further shown by Fahnestock and Rich. They demonstrated that the translational machinery could synthesize oligomers containing multiple ester bonds using chemically converted hydroxyPhe-tRNA^Phe^.^12^ However, each of these experiments was only possible because of an idiosyncratic route to the UAA-tRNA: chemical reduction of Cys, enzymatic charging of d-Tyr, and hydroxylation of the aromatic Phe residue. What was missing was a general method for producing the UAA-tRNA.

Right from the start there was significant interest in being able to incorporate biophysical probes into proteins using UAA mutagenesis. There were foundational approaches to the task of incorporating unique side chains. For instance, Johnson and colleagues modified Lys after it had been enzymatically ligated to tRNA. Acylation of the *N*_ε_-amine of Lys-tRNA^Lys^ with *N*-hydroxysuccinimide ester-azidobenzoic acid generated *N*_ε_-azidobenzoyl-Lys-tRNA^Lys^.^13^ Modified UAA-tRNA interfaced with endogenous translational machinery and was incorporated in place of or in competition with endogenous unmodified Lys in rabbit reticulocyte lysate. Because most target proteins contain multiple Lys residues, the modification could not be restricted to a single site, thus resulting in multisite incorporation of the photoactivatable Lys UAA.^13^

In 1978, Hecht and co-workers established a general procedure for the chemoenzymatic aminoacylation of tRNAs.^14^ T4 RNA ligase transfers an aminoacyladenylate moiety from N-blocked (with *o*-nitrophenylsulfenyl) aminoacylated *P*^1^,*P*^2^-bis(5′-adenosyl)diphosphates to tRNAs lacking the 3′-terminal adenosine. However, a large molar excess (>200-fold) of aminoacylated nucleotide derivatives were required for good yields, so they optimized the synthesis such that an only 20-fold molar excess was necessary, using *N*-acetylaminoacyl pCpA derivatives instead.^15^ The modified chemical aminoacylation was used to acylate tRNA^Phe^ with both d- and l-Phe, d- and l-Tyr, and *N*-acetyl-dl-β-Phe. Misacylated tRNAs can participate in peptide bond formation, consistent with the adaptor hypothesis, but efficient dipeptide formation with a poly-Phe message occurred primarily with l-Phe, l-Tyr, and, interestingly, β-Phe, with l-PhetRNA^Phe^ as the A-site tRNA.^15^

An alternative approach was demonstrated by Baldini and colleagues in 1988. Prior to this work, due to protection of the amino group during pCpA ligation, the chemically misacylated tRNAs could not bind the ribosomal A site and be incorporated into a growing polypeptide chain; thus, only dipeptides could form. By introducing a transient Boc protection/deprotection into the UAA-tRNA ligation, they demonstrated synthesis of functional *E. coli* tRNA^Phe^ charged with a photoactivatable cross-linker UAA, l-4′-[(3-trifluoromethyl)-3*H*-diazirin-3-yl]phenylalanine.^16^ However, protein yields were often low due to the stoichiometric nature of chemically acylated tRNAs, and modifications were limited because there were no general methods for UAA-tRNA synthesis. Johnson and Brunner’s methods both allowed more flexibility in the range of biophysical probes that could be attached to either Lys or Phe; however, they still were not general methods for UAA-tRNA synthesis, and they led to uncontrolled multisite incorporation of the biophysical probes.^13,16^

## A General Method for the Site-Specific Incorporation of UAAs *In Vitro*

**Figure 3:**
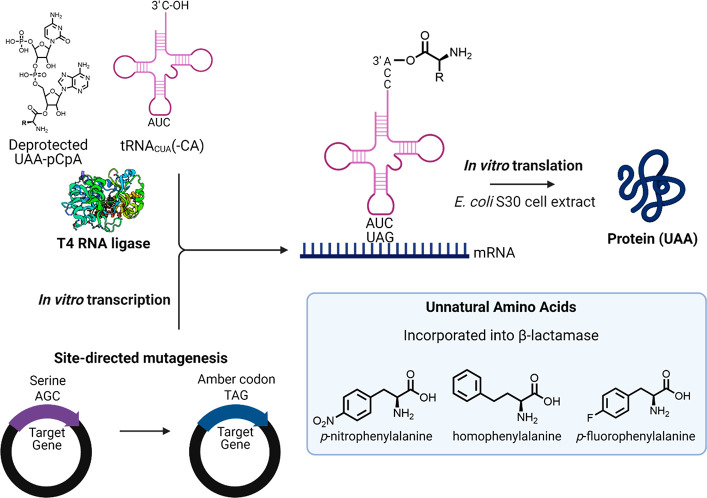
General method for site-specific UAA incorporation.

A breakthrough was the report of a general method for site-specific *in vitro* incorporation of UAAs by the Schultz lab (Figure 3).^17^ Briefly, they developed a general method for synthesizing UAA-tRNAs that recognized UAG stop codons and then demonstrated that this UAA-tRNA could be utilized by a crude *E. coli* S30 cell extract for site-specific incorporation of the UAA in response to a UAG codon engineered in a protein-coding gene.

The synthesis of the UAA-tRNA was made possible by an efficient chemical synthesis of the UAA-pdCpA, the two terminal nucleotides of the tRNA, and by technology previously developed by Sid Hecht and co-workers that showed UAA-pCpA molecules could be efficiently ligated to tRNA missing the terminal dinucleotide pCpA at the 3′-acceptor stem by the natural enzyme T4 RNA ligase.^18,19^ Brunner’s work enabling misacylated tRNAs to function in the ribosomal A site made it possible to form polypeptides and largely avoid hydrolysis of the amino acyl ester linkage by endogenous aaRSs.^16^ Drawbacks of the previous methods were nonselective incorporation of the UAA and size restrictions on the target protein. Noren’s UAA mutagenesis approach applied these foundational methods to a generalized system in which in theory a diverse range of UAAs could be used to acylate the suppressor tRNA, the suppressor tRNA could be directed to a specific site by mutagenizing that position to an amber stop codon, and the size of the protein of interest was limited only by what could be encoded on a plasmid.

Noren et al. (Schultz) showed the incorporation of three different UAAs in the active-site residue Phe66 in β-lactamase and kinetic characterization of these variants. They prepared an amber suppressor tRNA using anticodon loop replacement of yeast tRNA^Phe^ and demonstrated this tRNA was not recognized by the *E. coli* PheRS in their S30 extract [β-lactamase(Phe66TAG), non-acylated tRNA_CUA_, and [^3^H]Phe]. No β-lactamase activity was observed, and there was no band corresponding to [^3^H]Phe-incorporated β-lactamase by SDS-PAGE. Significantly, they showed using [^3^H]Phe-tRNA_CUA_ and HPLC analysis of trypsin-digested β-lactamase that the [^3^H]Phe was incorporated only at position Phe66, demonstrating not only efficient incorporation of the UAA [^3^H]Phe in response to the UAG codon but also that the UAA [^3^H]Phe was not scrambled with other natural amino acids.^17^

Bain and co-workers used a strategy similar to that of the Schultz group to incorporate l-3-iodo-tyrosine into a 16-residue polypeptide.^20^ They prepared a semisynthetic, non-hypermodified *E. coli* glycyl tRNA_CUA_ nonsense suppressor tRNA acylated with l-3-[^125^I]tyrosine and incubated with the message containing UAG at position 9 in rabbit reticulocyte lysate. The translation product was purified and sequenced to unambiguously determine the site specificity of incorporation. Nonsense suppression was due entirely to the added synthetic suppressor because they could not detect read-through by endogenous aminoacyl-tRNAs (aa-tRNA).^21^

### General Methods for UAA-tRNA Synthesis

**Figure 4:**
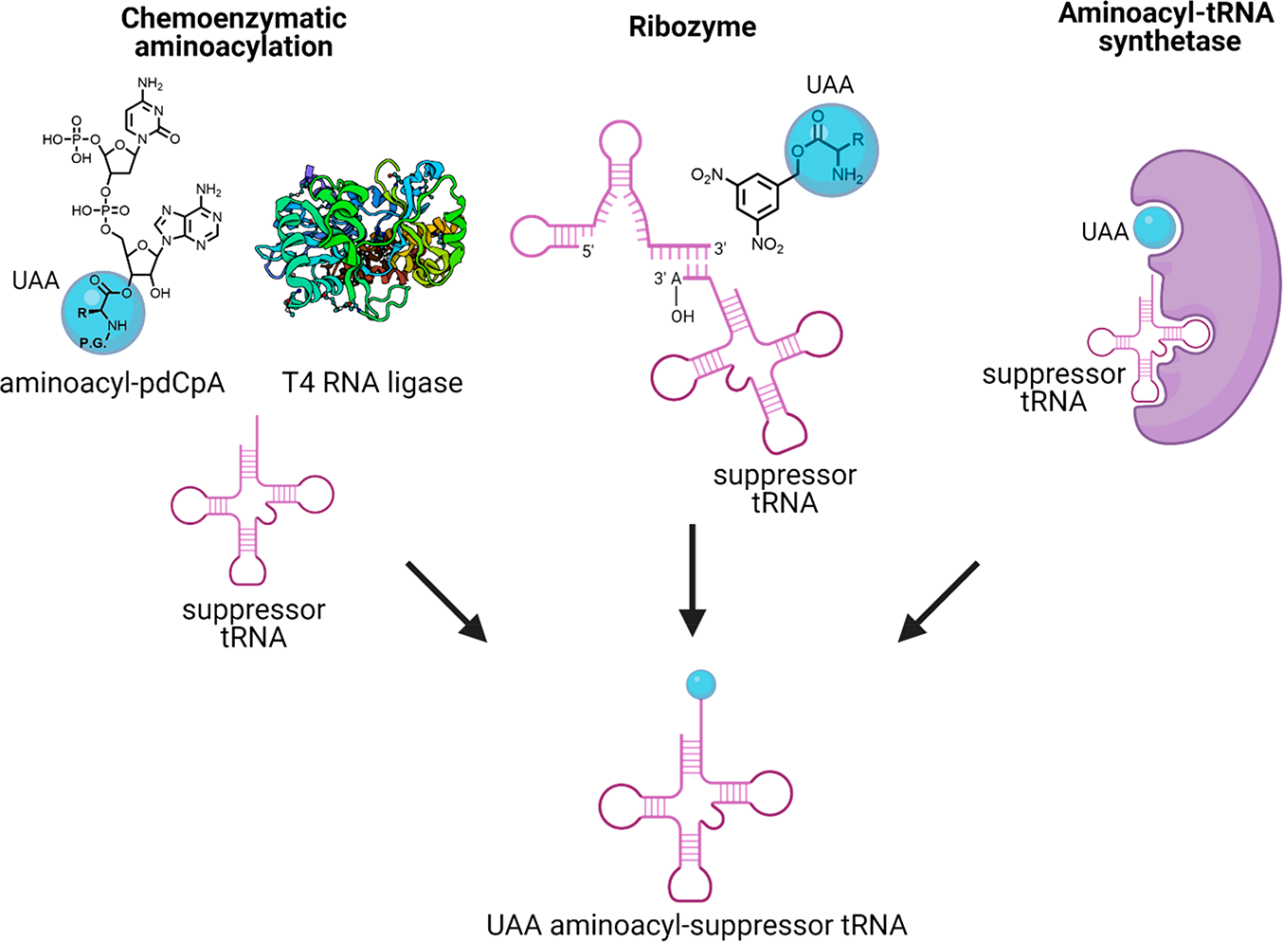
General methods for misacylating tRNA. P.G., protecting group.

A key issue continued to be the lack of a general method for synthesizing the UAA-pdCpA (Figure 4). A key advance was made by Robertson and Ellman (Schultz) in 1991.^22^ Unprotected pdCpA was selectively aminoacylated in high yield with the cyanomethyl ester (CME) of *N*-blocked amino acid and ligated to tRNA. The photolabile nitroveratryl protecting groups for the α-amine and side chain functional groups enabled the aa-tRNA to be deprotected photochemically. This reaction produces high yields of stable, unblocked aa-tRNA that can be used directly in a purified translation system. The approach greatly simplified the synthesis of UAA-pdCpA to one high-yield (76–87%) step.^22^

An alternative approach to tRNA aminoacylation was catalytic RNA, or ribozymes. Natural ribozymes catalyze trans-esterification reactions of phosphodiester bonds. Szostak and co-workers isolated catalytic RNAs with acyl transferase activity, like that of the PTC, from pools of random RNA sequences. They selected for enhanced transfer of an *N*-biotinyl-l-methionyl group from the 3′-end of a donor hexanucleotide, 5′-pCAACCA-3′, to the 5′-hydroxyl group of the ribozyme.^23,24^ Suga, Szostak, and co-workers generated aaRS-like ribozymes with two catalytic domains: one that recognizes the amino acid substrate and self-aminoacylates its 5′-hydroxyl and the other that binds the tRNA and transfers the aminoacyl group to the 3′-end. This ribozyme acts as a synthetase that can charge tRNA^fMet^ with Gln or Phe.^25^ CME was chosen as a leaving group on the amino acid because it has no hydrogen bond donors or acceptors that could interact with the ribozyme. Active RNAs could be isolated from the pool by selection with *N*-biotinyl-l-glutaminyl-CME and subsequent pull-down with streptavidin.^25^

The Suga lab generalized the ribozyme *de novo* catalyst for tRNA acylation using aaRS-like RNA molecules called Flexizymes (Fx) and mutants thereof.^26^ They noticed that Fx recognizes neither the leaving nor the amino group of the substrate, but rather the aromatic functionality of the amino acid side chain and the carbonyl group of the ester. To improve binding, they redesigned substrates incorporating an aromatic ring in the leaving group. They used dinitrobenzyl ester (DBE) and the more activated chlorobenzyl thioester (CBT). Because DBEs are less hydrolytically labile than CBTs, DBEs were used in further experiments. Enhanced interaction between Fx and the substrate significantly enhanced tRNA acylation efficiency and enabled incorporation of citrulline, *N*_ε_-acetyl-l-lysine, *N*_ε_-biotinyl-l-lysine, *p*-iodo-l-phenylalanine, (*S*)-3-isopropyllactic acid, and (*S*)-3-phenyllactic acid into short (nine-amino acid) FLAG-tagged peptides expressed in an *E. coli* cell-free translation system, Protein synthesis Using Recombinant Elements (PURE),^27^ by amber-programmed frameshift suppression and sense codon reassignment (AGU, AAC, and CAG).^26^ This breakthrough represented a powerful tool for enhancing the range of UAAs that could be ligated to tRNAs and incorporated into polypeptides *in vitro*.

### Expanding the Repertoire of UAAs to Address Questions of Protein Structure and Function

Another focus of the field following Noren et al. (Schultz) in 1989 was pushing the boundaries of what UAA structures and functions could be incorporated and using these UAAs to address important protein structure and function questions. Key questions of enzyme mechanism and protein stability could be addressed by incorporating unnatural isoelectronic or isosteric analogues of natural amino acids at sites of interest and measuring changes in enzyme kinetics and/or protein denaturation.

Building on earlier work with modified nucleosides and tRNAs, Ellman and Mendel working with Schultz explored the tolerance of the translational machinery to changes in the amino acid structure.^28^ Specifically, they tested if different UAAs could be incorporated at position Ala82 in T4 lysozyme (T4L) and how the UAAs affected the stability of T4L. Ala82 is a surface residue located between two helices, distant from the active site. The structure and electronics of the UAA significantly affected its use as a substrate by the translational machinery. The incorporation efficiencies were as follows: none detected (ND) for d-Ala and 30% (suppression efficiency) for lactic acid; *N*-alkyl amino acids, <5% for azetidine 2-carboxylic acid, 43% for pipecolic acid, 24% for *N*-methyl-alanine, and <5% for *N*-ethyl-alanine; α,α-disubstituted amino acids, 28% for cyclopropylglycine and 23% for α-aminoisobutyric acid. Interestingly, changing the amino acid structure and electronics changed the apparent yield of protein synthesis; because an *E. coli* S30 crude cell extract was used, the mechanism of this decrease in yield could not be determined at the time. The stabilities of the resulting UAA-substituted T4L proteins were determined by thermal denaturation as measured by circular dichroism.^28^ These UAA backbone analogues largely changed the stability of T4L as would be predicted.

Judice and Schultz used UAA incorporation to make more precise changes in amino acid structure than possible with the natural amino acids to probe enzyme mechanism.^29^ Staphylococcal nuclease (SNase) accelerates the hydrolysis of phosphodiester bonds in nucleic acids approximately 10^16^-fold. One hypothesis was that general base catalysis underpins this enormous rate acceleration where Glu43 in SNase acts as a general base to activate a water molecule for attack on the phosphodiester backbone of DNA. However, when Glu43 was replaced with isoelectronic and isosteric analogues, Arg, *S*-4-nitro-2-aminobutyric acid, *S*-2-amino-5-hydroxypentanoic acid, aminoethylhomocysteine, and citrulline, differing only by being poorer bases, the kinetics of SNase were virtually unchanged relative to those of the wild-type. A significant accomplishment at the time working with *E. coli* S30 cell extracts, a structure of the enzyme substituted with homoglutamic acid at site 43 was obtained. Combined, the kinetic and structural data suggested that Glu43 may instead play a structural role, fixing the conformation of a nearby loop.^29^

**Figure 5:**
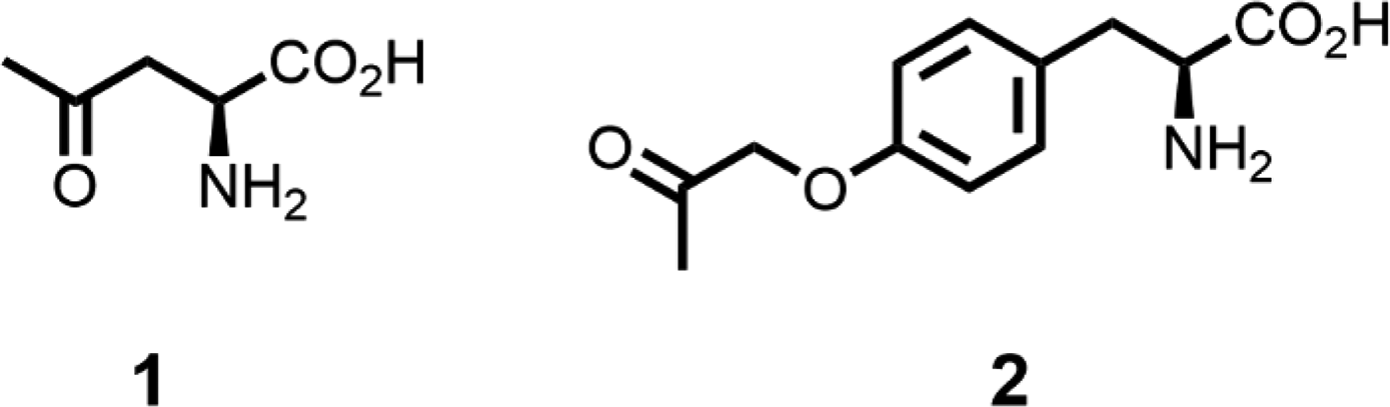
Ketone UAAs incorporated in T4 lysozyme in Cornish et al. (Schultz).^31^

Despite the enormous potential utility of biophysical and other probes, it was becoming clear that the translational machinery places constraints on the size of the amino acid side chain and hence what fluorophores, cross-linking agents, post-translational modification, or other UAAs could be incorporated.^30^ Thus, Cornish, Hahn, and Schultz incorporated a small ketone handle that could subsequently be modified to form an oxime or other unnatural linkage to the biophysical probe in what has come to be called bio-orthogonal labeling.^31^ They incorporated keto amino acids **1** (5% suppression efficiency) and **2** (30% suppression efficiency) in sites Ser44 and Ala82 in T4L, two sites known generally to give high suppression efficiencies (Figure 5). Subsequently, they showed that electrophilic ketone UAA **2** could be derivatized with fluorescein hydrazide in T4L Ala82 → **2**. Fluorescence spectra of purified T4L Ala82 → **2** and wild-type T4L both being subjected to the same labeling conditions with fluorescein hydrazide demonstrated that only the protein containing the ketone handle was labeled with the fluorophore.^31^

### Alternate Codons for UAA Incorporation

**Figure 6:**
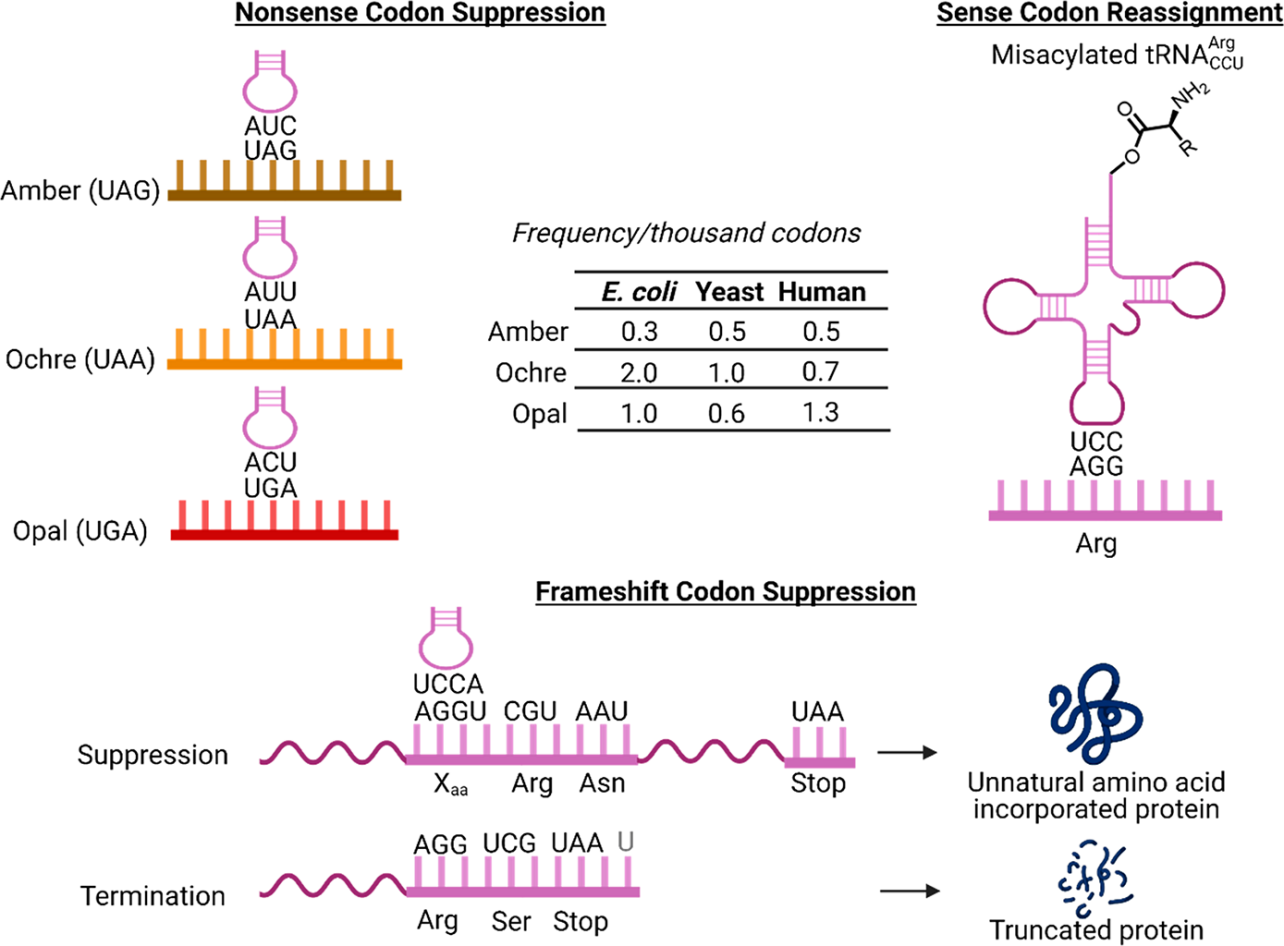
Alternate codons for UAA incorporation. Frequency data from https://www.genscript.com/tools/codon-frequency-table.

Another important area was exploring alternate codons for incorporation of UAAs. The main challenge of nonsense suppression is the limited range of nonsense codons: amber (TAG), ochre (TAA), and opal (TGA). Alternate codons would increase the number of UAAs that could be incorporated in a single protein. In the 1990s, frameshift codon suppression was explored as an alternative to nonsense suppression (Figure 6). When the frameshift does not happen, a termination codon UAA appears downstream, resulting in truncated protein. If the reading frame is shifted by suppression of the quadruplet codon with UAA-tRNA, full-length protein is synthesized.

Using this strategy, frameshift suppressor Ala-tRNA_ACCU_ and Ala-tRNA_CCUA_ incorporated Ala into *E. coli* dihydrofolate reductase (DHFR), shown by a restoration of enzyme activity to 40% and 15% of wild-type activity, respectively.^32^ Repurposing of rare codons was another strategy for expanding options for UAA incorporation. AGG was used as an alternative codon for incorporation of the photoactive UAAs *p*-phenylazophenylalanine, 2-anthrylalanine, 1-naphthylalanine, 2-naphthylalanine, and *p*-biphenylalanine into a polypeptide expressed in an *E. coli* S30 extract, where AGG is rare (<3%).^33^ The Sisido lab extended this work to incorporate the UAAs nitrophenylalanine, 2-naphthylalanine, *p*-phenylazophenylalanine, and 2-anthrylalanine into streptavidin (Tyr83 → AGGU) through frameshift suppression in *E. coli* S30 extracts.^34^ CGGG was found to work more efficiently than ACCU in further studies.^35,36^

More options for different quadruplet codons were demonstrated in an *E. coli* S30 cell extract with nitrophenylalanine efficiently incorporated into streptavidin using the codons AGGU, CGGU, CCCU, CUCU, CUAU, and GGGU.^37^ Further utility of this approach was demonstrated by the incorporation of two UAAs, nitrophenylalanine and 2-naphthylalanine, into streptavidin using the quadruplet codons CGGG and GGGU.^37^ Incorporation of two fluorescent UAAs in *E. coli* DHFR was achieved by a combination of quadruplet codon and amber suppression.^38^ Specifically, the fluorescent UAA 7-azatryptophan was incorporated with a CGGG quadruplet decoding tRNA and acceptor *N*_β_-dabcyl-1,2-diaminopropionic acid by amber suppression in *E. coli* DHFR.^38^

Sense codon reassignment, or genetic code reprogramming, has been explored as an alternative to nonsense suppression. Nonsense codon suppression is limited to two UAAs because there are only three nonsense codons and one must be used for translation termination. It would be of enormous practical utility and would allow fundamental questions about the genetic code to be addressed if sense codons could be reassigned. Forster, Tan, Cornish, and Blacklow established the concept of genetic code reprogramming of multiple, adjacent sense codons by reassigning three sense codons to UAAs using chemoenzymatically charged tRNAs in a reconstituted translation system lacking aaRSs.^39^

Rather than using an *E. coli* S30 *in vitro* extract with competing aa-tRNAs and aaRSs that could hydrolyze noncognate aa-tRNA pairs and recharge the tRNA with the cognate amino acid, we made a purified *in vitro* translation system ourselves based on published protocols in the ribosome mechanism field. The UAA-tRNAs were prepared using the chemoenzymatic methods being used by the Schultz group at the time. Because it was the first attempt to modify multiple sense codons, we started with three conservative side chain modifications: *O*-methyl-l-serine, 2-amino-4-pentenoic acid (allylglycine), and 2-amino-4-pentynoic acid (propargyl glycine). Tracking the peptide synthesis using [^35^S]Met-tRNA, [^3^H]Glu-tRNA, and authentic peptide markers prepared by solid-phase peptide synthesis, translation of a peptide with five of the same UAAs in a row was demonstrated. Finally, translation of a peptide with three different UAAs in a row each in response to a different sense codon was achieved. Together, this work showed for the first time that multiple sense codons could be reassigned allowing for translation of unnatural oligomers, a direction we argue below will become increasingly important to the field.

Around the same time, Josephson, Hartman, and Szostak established ribosomal synthesis of nonribosomal peptide-like molecules containing 10 UAA side chain analogues by sense codon reassignment, significantly increasing the number of UAAs that can be incorporated into a single polypeptide.^40^ The Suga lab established the mRNA-encoded incorporation of multiple, consecutive amino acid analogues for the *in vitro* synthesis of unnatural polypeptides using a combination of frameshift suppression and sense codon reassignment (AGU, AAC, and CAG) in the Fx/PURE reconstituted translation system with depleted aaRSs and cognate amino acids.^26^ The ability to control installation of multiple UAAs significantly increased the diversity of peptides that can be synthesized and screened for therapeutic properties. Suga has gone on to do a lot of work in this area, for example, incorporating multiple, consecutive amino acid backbone analogues into the peptide backbone, including α-hydroxy amino acids,^41^*N*_α_-methylated amino acids,^42^ and, more recently, d-amino acids^43^ and β-amino acids.^44^ However, to date, sense codon reassignment is limited to *in vitro* translation systems.

## A General Method for the Site-Specific Incorporation of UAAs *In Vivo*

To date, UAAs were incorporated into *E. coli* S30, wheat germ, or rabbit reticulocyte extracts. Methods developed in the laboratories of Dougherty and Lester pioneered the use of UAAs in *Xenopus* oocytes, where they injected UAA-pdCpA-ligated tRNA and mRNA encoding the protein of interest with an amber codon, with a particular focus on eukaryotic ion channels.^45^ Specifically, Lummis and co-workers explored the role of a highly conserved Pro at site 8 (Pro8) in cation-selective 5-hydroxytryptamine type 3 receptors. Pro8 acts as a hinge in the loop between the second and third transmembrane helices, a region that interacts with the extracellular ligand binding domain and was hypothesized to play an important role linking neurotransmitter binding to channel gating through *cis–trans* isomerization of the protein backbone. Incorporation of Pro analogues favoring the *cis* conformer produced functional channels, while those favoring the *trans* conformer produced nonfunctional channels. Importantly, the *cis–trans* energy gap of the Pro analogue was strongly correlated with channel activation, suggesting *cis–trans* isomerization of this single Pro acts as a gating switch between open and closed channel states.^45^ These experiments built upon their earlier work optimizing the pdCpA ligation chemistry for backbone analogues, such as α-hydroxy amino acids.^46^

With respect to expanding the repertoire of codons for incorporation of multiple UAAs in *Xenopus*, Rodriguez and co-workers demonstrated multisite incorporation of UAAs into nicotinic acetylcholine receptors by combining nonsense and frameshift codon suppression.^47^ A limitation of chemoenzymatically charged tRNAs is that they cannot be reacylated once inside the cell, capping the amount of protein that can be generated. An ideal system would include all of the necessary components genetically encoded in the cell. A main challenge of eukaryotic genetic code expansion is the fact that translational machinery is not well conserved between prokaryotes and eukaryotes.

The next big breakthrough in the field was full genetic encoding of UAA incorporation components in live cells. In 2001, Wang et al. (Schultz) reported a general method for incorporating UAAs into proteins in *E. coli* through directed evolution of an orthogonal aaRS/tRNA pair.^48^ This advance addressed multiple technical challenges in the field. One, it addressed the technical difficulty of preparing the aminoacyl-tRNA chemoenzymatically. Two, it removed the limit on protein yield imposed by use of an *in vitro* translation extract. Significantly, the UAA technology could now be adopted by a non-expert in the technology.

The key conceptual advance was the positive and negative selection strategy for evolving the orthogonal tRNA and aaRS.^49^ The naturally orthogonal *Mj*tRNA_CUA_^Tyr^ and *Mj*TyrRS were used as the starting point. First, they used a negative selection based on suppression of a UAG codon in the toxic RNase Barnase in the absence of *Mj*TyrRS to select for *Mj*tRNA_CUA_^Tyr^ variants that could not be aminoacylated by the endogenous natural aaRS enzymes.^49^ Next, the winners were subject to a positive selection for *Mj*tRNA_CUA_^Tyr^ variants that could suppress amber mutations in β-lactamase in the presence of *Mj*TyrRS.^49^ Finally, to generate the orthogonal *Mj*TyrRS, *E. coli* cells were transformed with a library of *Mj*TyrRS genes and subjected to a positive selection for suppression of a UAG codon in chloramphenicol acetyltransferase (CAT). The library was subject to both positive (+UAA, +chloramphenicol) and negative selection (−UAA, +chloramphenicol) to yield pairs that incorporate the UAA but not natural amino acids in response to the UAG codon. They used the resulting orthogonal aaRS/tRNA pair to incorporate *O*-methyl-l-tyrosine into DHFR expressed in *E. coli*.^48^

While still limited by the need to purify the UAA-incorporated protein out of the cell, this work eliminated the problem of low protein yields with *in vitro* cell extracts and broadly enabled engineering UAA biosynthetic machinery in live cells.^48^ There were some hurdles in getting there, with different selection approaches being less successful.^50^ Once a general method for generating mutant aaRS-tRNA pairs was established, issues that needed to be addressed were how to generate new orthogonal aaRS-tRNA pairs for UAA incorporation in model systems where large mutant libraries cannot be made, such as mammalian cells, and the lack of suitable starter aaRS–tRNA pairs orthogonal in these systems. The transfer of *E. coli* TyrRS-tRNA_CUA_ and LeuRS-tRNA_CUA_ to yeast and mammalian cells was feasible; however, tRNA expression was a major hurdle at the time. The Wang lab developed a general method for expressing orthogonal tRNAs in mammalian cells using type 3 Pol III promoters, and this is the general method currently being used in the field for UAA incorporation in yeast, mammalian cells, and various animals.^51^ Another challenge was that the size of the aaRS binding pocket limited the stereochemical diversity of UAAs that could be incorporated. A breakthrough in the field was made by the Wang lab with the finding that mutation of the *Methanosarcina* PylRS binding pocket can generate more flexible substrate specificity for Phe and Tyr analogues with bulky conjugated rings or long azobenzene side chains.^52^

The Schultz lab demonstrated the first fully genetically encoded UAA incorporation system in eukaryotes when they incorporated UAAs in *Saccharomyces cerevisiae*.^53^ They exploited the fact that *E. coli* tyrosyl tRNA_CUA_ can be expressed in yeast and is a poor substrate for the *S. cerevisiae* aaRSs. They evolved TyrRS in yeast for incorporation of acetyl, benzoyl, azido, and iodo-Phe analogues, as well as *O*-methyl-l-tyrosine, into human superoxide dismutase.^53^ This system became the basis for directed evolution of aaRSs for UAAs that could be readily transferred to mammalian cells. Genetic encoding of orthogonal synthetase–tRNA pairs in mammalian cells and animals, including *Caenorhabditis elegans*, *Drosophila melanogaster*, and *Mus musculus*, followed suit.^54−60^

### Incorporation of UAAs into Mammalian Cells and Animals

As early as 2006, Tirrell and Schuman established metabolic labeling with click handle UAAs as a nonspecific, heterogeneous multisite UAA incorporation method for tagging newly synthesized proteins in mammalian cells.^61^ Azidohomoalanine (AHA) is an azide-bearing methionine analogue that can be incorporated at methionine codons and tagged with an alkyne-affinity tag using copper-catalyzed azide-alkyne [3+2] cycloaddition for the identification of AHA-labeled proteins via mass spectrometry.^61^ Another methionine surrogate, alkyne-bearing homopropargylglycine (HPG), was used in tandem with AHA to fluorescently label newly synthesized proteins in rat hippocampal neurons by strain-promoted cycloaddition.^62^ Pulse-chase experiments enabled fluorescent labeling of two distinct proteomes synthesized sequentially in time such that the dynamics of protein synthesis and fate could be monitored in neurons.^62^ Thus, they could address important questions about the role of newly synthesized proteins in neuron function without the need for selective incorporation of the UAA.

Efficient incorporation of UAAs into proteins in animals has also been a critical challenge. To improve the efficiency, many different methods have been researched, including using the type 3 polymerase III promoter to more efficiently express orthogonal prokaryotic tRNAs,^51^ UAA esterification to increase UAA bioavailability,^63^ and optimizing tRNA/synthetase affinity to increase the level of UAA incorporation.^64^ Encouraging progress was achieved in this research area through the combined use of these optimized methods.^4^ In their letter to the editor of *Cell Research*, Ye, Wang, Li and co-workers reported the introduction, maintenance, and transmission of the genetic material for code expansion in mice. In this work, they integrated the orthogonal pAzFRS/tRNA_CUA_ pair into the mouse genome.^59^ They demonstrated that, in the presence of pAzF, the suppressor tRNA can decode the UAG amber codon to express a dual fluorescent reporter eGFP-TAG-mCherry in neurons and bone marrow cells of mice.^59^

### Expanding the Repertoire of UAAs That Can Be Incorporated *In Vivo*

One of the most exciting classes of UAAs continues to be backbone analogues because of the potential to extend the power of genetic encoding to oligomers other than α-l-polypeptides. The logic of making backbone analogue UAA incorporation work *in vivo* began with the discovery or generation of ribosomes that can accommodate these UAAs followed by testing of known aaRS/tRNA pairs for charging them. In 2016, Schepartz and Söll incorporated β^3^-amino acids into full-length DHFR in *E. coli*.^65^ Previously, Dedkova and Hecht had found that ribosomes from some erythromycin-resistant *E. coli* mutants could tolerate the incorporation of β^3^-amino acids into full-length DHFR *in vitro*.^66^ Building on this work, Schepartz and Söll highlighted the unexpected flexibility of the endogenous *E. coli* translational machinery to β^3^-amino acid backbone analogues when they demonstrated incorporation of β^3^-amino acids into DHFR expressed in *E. coli* harboring a plasmid encoding mutant ribosomes from erythromycin-resistant strains.^65^ Significantly, they demonstrated that β^3^-(*p*-Br)Phe and β^3^-Gly could be charged by endogenous aaRS enzymes, with PheRS being the most tolerant of these substrates. Furthermore, wild-type EF-Tu interacted efficiently with β^3^-Phe-tRNA^Phe^ for delivery to the ribosome. To improve the efficiency and selectivity for β^3^-amino acid incorporation, a library of peptidyl transferase center 23S rRNA mutant ribosomes were screened for erythromycin resistance and β-puromycin sensitivity, resulting in a new mutant P7A7 that imparted 3-fold higher levels of β^3^-amino acid incorporation over those of the previously discovered mutants.^65^ Tryptic digest of DHFR peptide fragments containing either α-Phe or β^3^-(*p*-Br)Phe at F128 showed a 10-fold lower level of incorporation of the β^3^-amino acid versus α-Phe.^65^

Underscoring the importance of recent work incorporating backbone analogue UAAs *in vivo*, it has been difficult for scientists to produce peptides containing d- and β-amino acids by UAA incorporation *in vitro*. By tuning tRNA sequence and concentrations of native initiation (IF_2_) and elongation factors (EF-Tu/Ts and EF-G), in 2017, the Suga lab increased the yield of a d-Ala-d-Ala-containing peptide by >5-fold and incorporated 10 consecutive d-Ser residues into a peptide chain.^43^ The existence of mutant *E. coli* ribosomes that enhance d-amino acid incorporation *in vitro* indicates potential for *in vivo* incorporation of d-amino acids.^67^ Similarly, in 2018, the Murakami lab improved incorporation of multiple β-amino acids, producing peptides with natural amino acid spacers between two or three β-amino acids in their optimized translation system.^68^ Translating the *in vitro* advances in incorporation of backbone analogue UAAs to cells should catch on as more suitable aaRS/tRNA pairs are established. In 2019, Dedkova and Hecht found that wild-type PylRS could incorporate a fluorescent oxazole UAA lacking an asymmetric center or α-amino group. MreB (Leu13TAG) was co-expressed with PylRS in an *E. coli* strain with modified ribosomes that could incorporate dipeptides and dipeptidomimetics.^66,69^ It is becoming clearer how malleable the translational machinery is for incorporation of more exotic UAAs. Importantly, this research signals the possibility of finding more mutant ribosomes and aaRS/tRNA pairs for backbone analogue UAA incorporation *in vivo*. One direction the field is going is to combine an expanded pool of UAA backbone analogues with an expanded pool of orthogonal codons and engineered orthogonal ribosomes for genetic encoding of unnatural oligomers *in vivo*.

Another class of UAA analogues that could be highly impactful is epigenetic protein modifications. Research on the functional effects of specific epigenetic protein modifications is hindered by the difficulty of synthesizing post-translationally modified target proteins in cells. *O*-phosphoserine (Sep) is the most abundant phosphorylated amino acid in eukaryotes. It is synthesized post-translationally by acylation of tRNA^Cys^ with Sep by SepRS, an aaRS unique to methanogenic archaebacteria. Park et al. (Söll, Noren, and Rinehart) made an amber suppressing tRNA^Sep^ and, critically, evolved an EF-Tu mutant, EF-Sep, that could bind Sep-tRNA^Sep^ for site-specific incorporation of Sep into proteins in *E. coli*.^70^ SepRS, tRNA^Sep^, and EF-Sep together allow *E. coli* to read UAG as a Sep codon; they synthesized mitogen-activated ERK activating kinase 1 (MEK1) with Sep incorporated at a key modified residue Ser218 by amber suppression *in vivo*.^70^ In addition, it would need to be established that Sep was incorporated at only the intended UAG residue. Similarly, tyrosine phosphorylation is a critical PTM in cellular signal transduction. In 2017, Luo et al. (Schultz and Wang) incorporated *O*-phosphotyrosine (pTyr) and its nonhydrolyzable analogue, 4-phosphomethylphenylalanine, into recombinant proteins by amber suppression in *E. coli*.^71^ Around the same time, Hoppmann and co-workers (Hunter, Shokat, and Wang) incorporated a neutral pTyr analogue into recombinant proteins in *E. coli* by amber suppression; deprotection results in a native, negatively charged pTyr at the desired site.^72^ Multisite incorporation of these UAAs would enable modeling of multiple phosphorylated residues of a protein.^71,72^

Low yield is a significant hurdle to studying proteins modified with UAAs by amber suppression *in vivo*. Wild-type expression levels of UAA-modified protein cannot be realized because suppressor tRNAs compete with release factors (RFs) for the stop codon. Church, Isaacs, and co-workers have pioneered the breakthrough in this area by developing genomically recoded organisms (GROs). Upon recoding of the entire genome of *E. coli* such that all UAG stop codons are mutated to the UAA stop codon and deletion of RF1, the “blank” UAG codon could then be reintroduced as a sense codon for highly efficient incorporation of a UAA.^73^ The new genome enabled this new strain of *E. coli* C321.ΔA to exhibit increased resistance to viral infection by blocking the translation of viral proteins.^73^ The Church lab has led this area of research toward creating GROs with expanded capabilities. Ostrov and co-workers (Church) created *E. coli* with a 57-codon genome in which all 62214 instances of seven codons were replaced with synonymous codons in all protein-encoding genes.^74^ When the recoded codons’ respective tRNAs and release factor are removed, up to four orthogonal UAAs could be introduced into the organism.^74^ The increased yield and specificity of UAA incorporation in GROs should empower efforts for industrial UAA-modified protein production and more representative *in vivo* experiments with UAA-modified proteins.

The genomically recoded *E. coli* strain C321.ΔA has been used subsequently to advance techniques for studying post-translational modifications with UAAs. Isaacs and Rinehart conducted a proteome-wide investigation of the role of phosphorylation of human proteins *in vivo*.^75^ They genetically encoded Sep^70^ in a synthetic human phosphopeptide library expressed in C321.ΔA and identified proteome-wide phosphorylation-dependent interactions using bimolecular fluorescence complementation in cells.^75^ In contrast, for *in vitro* studies of the regulation by phosphorylation of 26S proteasome subunit RpnI, Sep was incorporated into RpnI(361TAG) expressed in *E. coli* strain C321.ΔA, allowing for purification of homogeneously phosphorylated RpnI.^76^ To transfer this technology to mammalian cells, Chin and co-workers demonstrated orthogonality in mammalian cells of an evolved SepRS/tRNA_CUA_ pair^77^ based on the original by Park and co-workers.^70,78^ The Sep incorporation system in mammalian cells was completed by co-expression with eRF1(E55D) (a eukaryotic RF more permissive of UAG read-through), creation of a eukaryotic elongation factor variant EF-1α-Sep containing mutations analogous to those of the prokaryotic EF-Sep, and deletion of phosphoserine phosphatase to increase the intracellular Sep concentration.^78^ If the UAA technology can be used to selectively introduce Sep (and ultimately other epigenetic modifications simultaneously) at multiple, defined positions in a single protein in living cells, this will be a very powerful tool for biologists.

**Figure 7:**
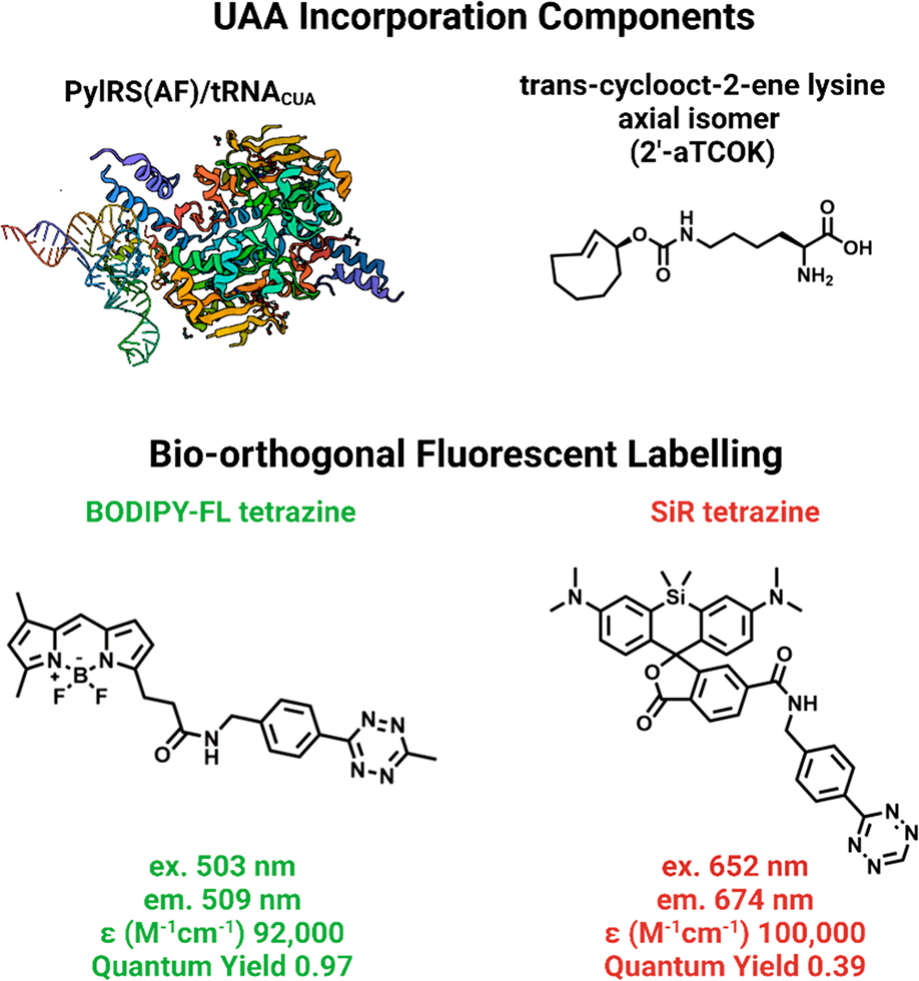
Ideal UAA incorporation components and fluorescent click reagents for bio-orthogonal intracellular protein labeling in mammalian cells.

Fluorescent UAAs remain one of the most sought after yet challenging classes of analogues. Tagging with fluorescent proteins (FP) is an indispensable technique for localization and mechanistic studies of protein targets inside cells. However, the large size of FPs (27 kDa) limits tagging to the protein termini and the targets that can be studied. The most efficient chemical tags also require protein tags, and the peptide chemical tags rely on two-step labeling with bio-orthogonal chemistry. The holy grail of fluorescent labeling would be direct and selective incorporation of a fluorescent UAA with a high photon output in mammalian cells. The challenges are having the ribosome accept a large fluorophore as a substrate, minimal nonspecific labeling of the fluorescent amino acid in the cell or incorporation into other UAG codons, or rapid and selective bio-orthogonal chemistry; one indication that the labeling technology meets these criteria is that it can be used to image a typical cellular protein at ∼1 μM concentrations freely diffusing intracellularly with single-molecule resolution. Comprehensive reviews of this literature have been published,^79,80^ and we have highlighted advances in this field and the related chemical tagging field previously.^81^ Here we focus on the significant challenge of efficient and robust fluorescent labeling of intracellular proteins in mammalian cells, highlighting the systematic comparison of fluorescent UAA labeling by bio-orthogonal click chemistry technologies from Peng and Hang.^82^ While there has been success in labeling intracellular proteins in live mammalian cells using bio-orthogonal strain-promoted click chemistry,^83,84^ the majority of published work still focuses on cell surface proteins. Peng and Hang wanted to observe the localization and trafficking of the small membrane-associated protein interferon-inducible transmembrane protein 3 (IFITM3) in cells. Given that GFP is twice the size of IFITM3, they took advantage of the small size and modularity of organic fluorophores afforded by the UAA technology. Site-specific incorporation of the commercially available *trans*-cyclooct-2-ene lysine (2′-aTCOK) into IFITM3 by amber suppression with the *Mm*PylRS(Y306A, Y384F)AF/tRNA pair^85−87^ led to the most efficient and specific labeling with monosubstituted tetrazine (Tz) fluorophores, e.g., tetrazine silicon rhodamine (H-Tz-SiR), in live mammalian cells (Figure 7).^82^ H-Tz-SiR was the best all around for intracellular labeling; H-Tz-BODIPY-FL was good but is more appropriate for membrane protein labeling due to its relative hydrophobicity.^82^

The alternative to bio-orthogonal click chemistry labeling is direct incorporation of a fluorescent UAA. The benefit of this approach is ease. All that is required is transfection of the aaRS/tRNA and target constructs, incubation with the UAA, and washing out excess UAA. The drawbacks are the limitations on the size of the fluorescent side chain due to the aaRS binding pocket and, thus, the tendency for these fluorophores to be relatively blue-shifted and dim. Nonetheless, site-specific fluorescent labeling of proteins with fluorescent UAAs is built on the foundation of direct incorporation of a single fluorescent amino acid. Jan and Cohen established the state of the art with their incorporation of environmentally sensitive fluorescent UAA Aladan into the B1 domain of streptococcal protein G (GB1) by solid-phase synthesis.^88^ The small size, the flexibility of the incorporation site, and the keen environmental sensitivity of Aladan provide unparalleled spatial resolution for detecting the electrostatic properties of various regions of this protein.^88^

*In vivo* incorporation of Aladan analogues, such as Anap, was enabled by the establishment of aaRS/tRNA pairs for this UAA, derived from *E. coli* LeuRS/tRNA_CUA_.^89^ Anap undergoes a blue-shift in fluorescence emission in increasingly hydrophobic environments, making it promising as a sensor of protein conformational change. Shandell, Cornish, and Kass demonstrated the feasibility of sensing the conformational change of a population of UAA-modified cardiac voltage-gated sodium channels expressed in live mammalian cells through incorporation of Anap into the inactivation gate, a dynamic ∼50-amino acid intracellular linker.^90^ Since the foundational work of Dougherty and Lester, ion channel physiologists have embraced the UAA technology in oocytes.^91,92^ Significantly, Puljung and co-workers incorporated Anap into K_ATP_ channels in live mammalian cells, enabling voltage-clamp fluorimetry experiments in this new, possibly more physiologically relevant context.^93^ Ligand binding or conformational change measured by Anap environmental sensitivity can now be coupled to functional changes in channel gating measured by electrophysiology in mammalian cells.^93^ More impactful applications of the technology are being published as technical challenges are overcome and efficiency and ease of use improved. When multiple fluorophores with single-molecule resolution can be incorporated selectively into mammalian cells, the UAA technology will be a powerful tool for studying biological mechanism in living cells.^94^

## New Conceptual Applications of UAA Incorporation

Wonderfully creative, the UAA technology will inspire myriad new directions. One particularly expansive direction is to engineer not only the amino acids but also the nucleic acids, organelles, and other parts of the cell to give rise to unnatural, chimeric, and semisynthetic organisms (SSOs). In some ways, this is the counterpart to building an artificial cell ground up from artificial RNA parts.^95^ To model biological conditions that could explain the transition from an RNA world, Schultz and co-workers engineered chimeric *E. coli* in which 40% ribonucleotide versus deoxyribonucleotide could be incorporated into the genome when the size of the pool of deoxyribonucleotide triphosphates in the cell was significantly decreased in concert with defects in DNA repair.^96^ In a similar fashion, Schultz and co-workers modeled the central hypothesis of endosymbiotic theory that mitochondria could have evolved from prokaryotes entering host cells and being maintained as endosymbionts. They engineered chimeras of *E. coli* and *S. cerevisiae* in which mutant *E. coli* live in the cytosol of and provide ATP to a respiration-deficient yeast mutant or yeast provide thiamin to a resident *E. coli* thiamin auxotroph.^97^ SSOs can be generated by codons containing unnatural base pairs or through the engineering of sense codon usage. Zhang et al. described an optimized SSO that stores genetic information using DNA containing two additional letters, which form a third, unnatural base pair, dNaM (mRNA codon) and dTPT3 (tRNA anticodon).^98^ This expanded genetic code enables decoding of new codons to direct site-specific incorporation of UAAs into proteins in *E. coli*.^98^ Recently, the Chin lab announced the creation of a SSO with a 61-codon genome. Creation of such a synthetic, minimally recoded *E. coli* genome by compressing synonymous codons addresses origins of life questions and biological mechanism and is enormously useful for therapeutic applications.^99^

### Mechanistic Studies of Translation with UAAs

Somewhat surprisingly, there has been little integration of the incredible advances in our understanding of the structure and mechanism of the translational machinery and the UAA technology since the breakthrough publication by Noren et al. (Schultz) in 1989. It has often been thought that natural limitations of the translational machinery underlie the difficulty in incorporation of d- and β-amino acids. It turned out that the translational machinery may be more tolerant to unnatural substrates than previously thought. Mechanistic insight into how these analogues interfere with translational machinery was needed. Leyh, Gonzalez, Cornish, and co-workers further clarified the mechanism by which d-amino acids disrupt translation in a purified translation system, finding that while d-aa-tRNA can be accepted at the A site, act as a peptidyl-transfer acceptor, and translocate the peptidyl-d-aa-tRNA into the P site, this process occurs slowly.^100^ Furthermore, the peptidyl-d-aa-tRNA at the P site partitions ribosomes into arrested and non-arrested subpopulations. Chemical protection and molecular dynamics simulations demonstrated that P-site-bound peptidyl-d-aa-tRNA traps the PTC in a conformation that is not conducive to peptidyl transfer, providing insight into how the ribosome discriminates between l- and d-amino acids.^100^ This mechanism of discrimination against d-amino acids appears to be similar to other peptide stalling mechanisms of the ribosome and may suggest the mechanism by which it discriminates against UAAs generally. Further mechanistic work with UAAs no doubt would lead to additional mechanistic surprises and could significantly inform efforts to improve the efficiency and breadth of the technology.

### Engineering Multisite UAA Incorporation *In Vivo*

Whether the objective is to incorporate different epigenetic modifications or to synthesize an unnatural backbone oligomer, it will be critical to be able to incorporate multiple different UAAs *in vivo* using alternate codons. Yields of UAA-incorporated proteins expressed in mammalian cells are significantly lower than those expressed in *E. coli*. Thus, it is an uphill battle to yield multiply UAA incorporated proteins in mammalian cells. In 2013, Chatterjee et al. (Schultz) developed a baculovirus-based delivery system for efficient incorporation of UAAs into proteins in mammalian cells.^101^ Later that year, the same mammalian suppression system was applied to incorporate two distinct UAAs (eBK and OMeY) into EGFP in HEK293T cells using TAG and TAA suppression with *Ec*TyrRS/tRNA_CUA_ and *Mb*PylRS/*Mm*tRNA_UUA_ pairs, respectively. They also demonstrated the application of dual suppression to fluorescent labeling of antibody–drug conjugates (anti-Her2-IgG-nAF) purified from HEK293F cells.^102^ In mammalian cells, nonsense suppression suffers from a low level of expression of suppressor tRNAs and competition with endogenous release factors seeking to truncate target protein. In 2011, Johnson and co-workers established that knockout of release factor 1 in *E. coli* enables incorporation of UAAs at multiple TAG sites in the same gene.^103^ In mammalian cells, by optimizing the PylRS/tRNA_CUA_ expression system and engineering eukaryotic release factor 1, Schmied and co-workers were able to increase the yield of protein containing UAAs at three sites by 2–4-fold.^104^ Multisite incorporation at unique codons selectively *in vivo* remains a challenge for the field.

### Engineering the Ribosome for Improved Incorporation of UAAs

One strategy toward multisite UAA incorporation at unique codons is to take advantage of our growing structural and mechanistic understanding of the translational machinery to engineer the ribosome itself. When the toolbox is expanded to include both quadruplet and amber codons, the ability of the natural ribosome to decode such codons limits the efficiency of UAA incorporation and the resulting yield of UAA-incorporated protein. It has been an outstanding challenge incorporating multiple UAAs in a single protein even in *E. coli* expression systems due to the lack of several blank codons and mutually orthogonal aaRS/tRNA pairs. In a breakout publication, Rackham and Chin addressed this challenge by engineering an orthogonal ribosome in *E. coli* via an engineered duplicate, orthogonal Shine-Dalgarno mRNA sequence/16S small ribosomal subunit RNA pair.^105^ Building on this work, Chin and co-workers have evolved an orthogonal ribosome for quadruplet and amber decoding.^106^ Orthogonal ribosomes can be devoted to efficient decoding of alternative codons because they are directly targeted to a corresponding orthogonal mRNA and thus do not synthesize the proteome. Through the combined use of mutually orthogonal aaRS/tRNA pairs, the evolved ribosome, and corresponding orthogonal mRNA, two UAAs were incorporated into single recombinant fusion proteins in *E. coli*. The cross-linking UAA *p*-benzoylphenylalanine (Bpa) and click handle pAzF were incorporated into glutathione *S*-transferase (GST)-maltose binding protein in response to a quadruplet and amber codon. The alkyne *N*6-[(2-propynyloxy)carbonyl]lysine (CAK) and pAzF were incorporated into GST-calmodulin in response to a quadruplet and amber codon.^106^ Incorporation of pAzF and CAK into calmodulin enabled formation of a triazole cross-link by copper-catalyzed click chemistry, demonstrating precise genetic control of protein conformation with the UAA technology.

**Figure 8:**
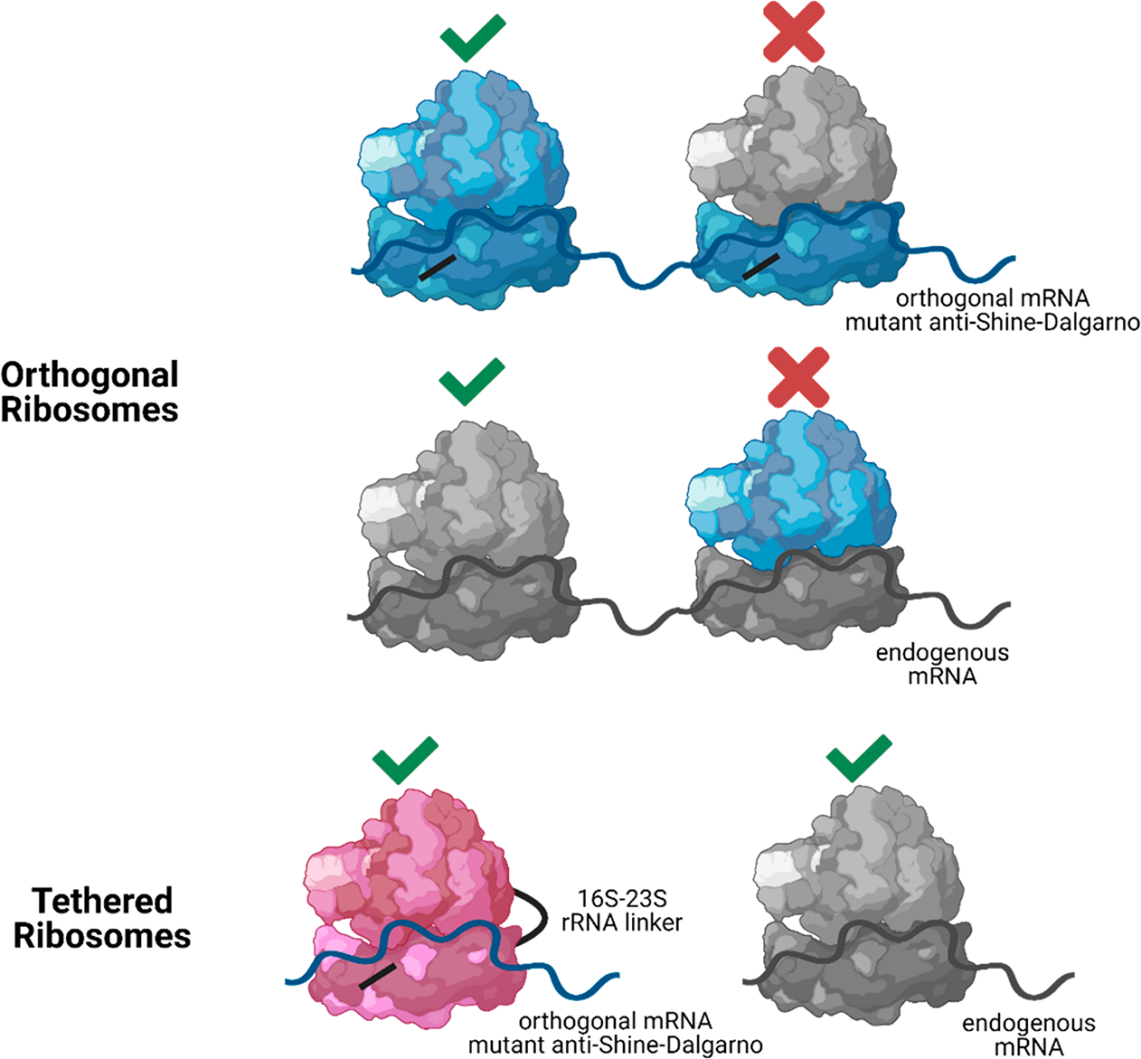
Bio-orthogonal translation with orthogonal and tethered ribosomes.

Early strategies for achieving orthogonality involved the development of orthogonal aaRS/tRNA pairs, building of orthogonal genetic codes, and creation of orthogonal ribosome–mRNA pairs by engineering the 16S rRNA and complementary mRNA Shine-Dalgarno sequences.^107^ However, the continuous exchange of the subunits of the ribosome still limits the establishment of complete orthogonality with native translation (Figure 8). To address this gap, Jewett and Mankin used a circular permutation approach to systematically generate linked 16S–23S rRNA variants that could assemble functional tethered ribosomes in cells.^108^ They demonstrated that the engineered ribosome with inseparable tethered subunits (Ribo-T) is capable of supporting the growth of *E. coli* cells, wholly orthogonal, and does not interfere with wild-type ribosomes.^108,109^ They demonstrated the unique utility of Ribo-T in studying dominant lethal mutations of rRNA, a nearly impossible task in other cell models.^108^ The Chin lab used a similar approach to covalently link the small and large ribosomal subunits by RNA linkers.^110,111^ The compatibility of the tethered ribosomal complexes with the multisite incorporation of UAAs was evaluated by Jewett and Mankin through the fluorescence analysis of a super folder GFP (sfGFP) variant containing five TAG codons, finding that the tethered translation system is effective in incorporating five pAzF click handles into sfGFP.^112^ What remains is to show incorporation of multiple different UAAs in a row, each in response to a different codon.

### Application of UAA Technology to Biomedicine

The UAA technology is ideal for antibody–drug conjugate (ADC) generation and other applications in biomedicine. The problem is production of homogeneous titers of ADCs for targeted cancer chemotherapy. ADCs are anticancer therapies designed to target tumors with cytotoxic small molecule drugs. Bypassing normal tissues reduces the toxic side effects of chemotherapy. Site-specific conjugation of the small molecule drug to the antibody homogenizes the mixture of ADCs, thus providing more reliable pharmacokinetic properties, efficacy, and safety profiles. In 2014, Tian and co-workers produced gram per liter scale titers of UAA-incorporated ADCs from stable CHO cells using antibodies targeting common antigens on colorectal/gastric and breast cancers.^113^ Ambrx, Inc., developed an anti-HER2 ADC product ARX788 using the UAA incorporation strategy. ARX788 is generated by the formation of a highly stable oxime bond between a noncleavable Amberstatin (AS269) drug linker and a ketone-bearing UAA, pAcF, which was incorporated into the primary sequence of the antibody through amber codon suppression. The Food and Drug Administration has granted fast track designation to ARX788 for the treatment of patients with advanced or metastatic HER2-positive breast cancer in 2021.^114^

UAAs can also be used for vaccine development. In 2014, Wang and co-workers developed a theoretically safe and effective HIV-1 vaccine by making viral replication dependent on the presence of UAA and the aaRS/tRNA pair.^115^ In 2016, the Zhou lab developed a live attenuated influenza A vaccine strain containing multiple amber codons in its genome.^116^ The strain can be replicated only in a transgenic 293T cell line that harbors an orthogonal amber suppressor aaRS/tRNA_CUA_ pair and the cognate UAA, making it replication incompetent in normal human cells and thus useful for immunization.^116^ Undoubtedly, the UAA technology can be exploited in other modalities for therapeutics.

### Studying Fundamental Biological Processes with UAA Technology

Once multiple different UAAs can be incorporated at unique codons *in vivo* with no cross reactivity, the UAA technology will be a powerful tool for systems biology that enables biological mechanism to be studied in living cells. As an example, biologists would like to control various protein signaling states by turning on and off receptor–ligand interactions and intraprotein interactions. Such control would enable clear-cut conclusions about the functional effects of specific protein structures in the native cell environment. This was recently illustrated in a study by the Chen lab exploiting transition metal-based bio-orthogonal cleavage reactions for on-demand release of toxic drugs from ADCs and precise control of ligand–receptor interactions at the cell surface.^117^ In this study, they used the genetic code expansion strategy to incorporate chemically caged Tyr and Lys analogues into eight different sites of Z_Her2_, a small protein with a high affinity for the membrane protein HER2. The UAA-modified Z_Her2_ mutants were expressed in *E. coli*, purified, and fluorescently labeled. Fluorescent Z_Her2_ and its UAA-modified mutants were incubated with SK-BR-3 cells, a human breast cancer cell line that overexpresses HER2, and analyzed by flow cytometry. Strong fluorescence was observed when wild-type Z_Her2_ and HER2 interacted. A decrease in fluorescence was observed with caged UAA-modified Z_Her2_ mutants that could no longer interact. Fluorescence was rescued upon decaging to the native amino acid. This allowed them to directly probe the functional role of each amino acid residue in the interaction between Z_Her2_ and Her2.^117^ It should be emphasized that the UAA technology is at a sufficiently mature stage where it can be adopted by biologists not expert in the methodology with similar ease and with even more diverse applications, perhaps, than GFP.

## Future Directions

Considerable progress has been made in the field of genetic code expansion since the publication of the study by Chatterjee et al. in 2013. However, many technical challenges remain, and the application of the technology to drug development and basic science is only just beginning.

Significantly changing the structure of the amino acid and still having it be accepted as a substrate by the translational machinery remains difficult. For example, there are limitations to what backbone analogues can be incorporated for unnatural oligomer synthesis or powerful fluorophores for biophysical studies of proteins. There have been incredible advances in our understanding of ribosome structure and function since Schultz, Noren, and co-workers published their seminal paper in 1989. In collaboration, we demonstrated that this mechanistic knowledge could be exploited to gain insight into how the translational machinery discriminated the structure and electronics of the amino acid. More mechanistic work with UAAs is needed. This mechanistic work can provide insight into the mechanism of translation and guide future engineering efforts. It also is now possible to engineer translation and the ribosome at a scale not previously possible with GROs, stapled ribosomes *in vivo*, and a growing arsenal of UAAs. Together, these advances allow direct selection for orthogonal ribosomes that work with an expanded set of UAAs and codons.

Optimization has made expression and purification of UAA-incorporated proteins from *E. coli* a robust method accessible to scientists not expert in the field. Biochemists and biologists are encouraged to adopt the technology and apply it to a variety of mechanistic questions. With the ability to produce a broad range of UAA-containing proteins in *E. coli* in high yield and advances in bio-orthogonal labeling methods, the field is at an exciting moment to apply the technology to challenging problems in drug discovery and biotechnology. Antibody–drug conjugates and vaccine development likely are just the start for therapeutic applications of the technology. Undoubtedly, an important next step for the field will be synthesizing unnatural oligomers directly in *E. coli*.

The next challenge is to develop methods for incorporating multiple, different UAAs in response to different codons in live mammalian cells. This would allow mechanistic questions, like the role of different epigenetic modifications, to be addressed directly in the cell. Technically, this likely will require (1) using GROs so the UAAs are not incorporated at endogenous codons, (2) engineering tethered ribosomes in mammalian cells, and (3) evolving tethered ribosomes to work with different UAAs and different codons. If successful, like GFP before it, UAAs could be a powerful tool for studying biological mechanism in live cells, but with a much broader repertoire of chemical functionality.

## Conclusion

The incorporation of unnatural amino acids by the translational machinery using artificial UAA-tRNAs is a wonderfully creative idea bringing together organic chemistry, molecular biology, and synthetic biology of the translational machinery. It is now a robust field with many who trained in the technology leading their own exciting advances for the tools in the UAA repertoire, in the aaRS/tRNA orthogonal pairs, in moving to higher organisms, and even in tethered orthogonal ribosomes and *de novo* GROs. Looking forward, as the tools continue to progress, UAA mutagenesis no doubt will become an essential tool for asking fundamental questions in systems biology and will be further adapted as a new strategy for drug development.
